# Gut mycobiomes are altered in people with type 2 Diabetes Mellitus and Diabetic Retinopathy

**DOI:** 10.1371/journal.pone.0243077

**Published:** 2020-12-01

**Authors:** Rajagopalaboopathi Jayasudha, Taraprasad Das, Sama Kalyana Chakravarthy, Gumpili Sai Prashanthi, Archana Bhargava, Mudit Tyagi, Padmaja Kumari Rani, Rajeev Reddy Pappuru, Sisinthy Shivaji

**Affiliations:** 1 Jhaveri Microbiology Centre, Brien Holden Eye Research Centre, L. V. Prasad Eye Institute, Kallam Anji Reddy Campus, Hyderabad, Telangana, India; 2 Smt. Kanuri Santhamma Centre for Vitreo-retinal Diseases, L. V. Prasad Eye Institute, Kallam Anji Reddy Campus, Hyderabad, Telangana, India; 3 Internal medicine, L. V. Prasad Eye Institute, Kallam Anji Reddy Campus, Hyderabad, Telangana, India; University of Hyderabad, INDIA

## Abstract

Studies have documented dysbiosis in the gut mycobiome in people with Type 2 diabetes mellitus (T2DM). However, it is not known whether dysbiosis in the gut mycobiome of T2DM patients would be reflected in people with diabetic retinopathy (DR) and if so, is the observed mycobiome dysbiosis similar in people with T2DM and DR. Gut mycobiomes were generated from healthy controls (HC), people with T2DM and people with DR through Illumina sequencing of ITS2 region. Data were analysed using QIIME and R software. Dysbiotic changes were observed in people with T2DM and DR compared to HC at the phyla and genera level. Mycobiomes of HC, T2DM and DR could be discriminated by heat map analysis, Beta diversity analysis and LEfSE analysis. Spearman correlation of fungal genera indicated more negative correlation in HC compared to T2DM and DR mycobiomes. This study demonstrates dysbiosis in the gut mycobiomes in people with T2DM and DR compared to HC. These differences were significant both at the phyla and genera level between people with T2DM and DR as well. Such studies on mycobiomes may provide new insights and directions to identification of specific fungi associated with T2DM and DR and help developing novel therapies for Diabetes Mellitus and DR.

## Introduction

Dysbiosis, the alterations in diversity, abundance and functionality in the gut microbiome (especially bacteria and fungi) has been implicated in several diseases. Of the bacterial and fungal microbiomes, the former has been more extensively studied and demonstrated to be associated with auto-immune and inflammatory diseases [1–5], cancers and mental disorders [6, 7] and many ocular diseases [8–19]. There are increasing reports of dysbiotic changes in the gut fungal microbiomes (mycobiomes) in individuals with several disorders including Crohn’s disease, colitis, inflammatory bowel disease (IBD) and bowel syndrome, colorectal cancer, diarrhea, intestinal allografts, and alcoholic liver disease [20–36]. Mycobiome changes have also been recorded in immune-compromised hosts [37, 38] and in patients with diabetes mellitus (DM), obesity [39–41] and eating disorders like anorexia nervosa [42]. These studies reported an increase in *Candia* spp. (*C*. *tropicalis*, *C*. *glabrata*, *C*. *albicans*) in gut mycobiome of people with IBD and alcoholic liver disease; a decrease in *Saccharomyces cerevisiae* in IBD and increase in *Trichosporon* spp. and *Malassezia* spp. in colorectal cancer [27, 36, 43–45]. There are also indication of pathologic conversion of some commensal eukaryotes under certain disease process [46–48]. Dysbiosis in gut mycobiomes has also been implicated in diseases (like allergic pulmonary disease, hepatitis B cirrhosis, chronic hepatitis B and Rett syndrome) that are not gut-associated [21, 37, 49].

Diabetic Retinopathy (DR) is the most common blinding ophthalmic disorder in people with DM [50] and the prevalence of DR in Type 2 Diabetes Mellitus (T2DM) patients increased from 28.8% at less than five years to 77.8% after 15 or more years [51]. The International Diabetes Federation (IDF) has estimated that T2DM currently affects over 463 million people in the world and is expected to increase to 700 million in 2045 [52]. Earlier studies have documented dysbiosis in the gut bacterial microbiome [1, 2, 53] and mycobiome [40, 54, 55] in people with DM but did not find any difference between people with type 1 and 2 DM [40, 54, 55]. Further, it is not known whether dysbiosis in the gut mycobiome of T2DM would be reflected in people with DR.

The primary aim of the current study was to characterize and assess the gut mycobiome differences of individuals with T2DM, T2DM with DR and healthy controls. Such studies may provide new insights and directions to identification of specific fungi associated with T2DM and DR, and help developing novel therapies for treatment of DM and DR [56, 57].

## Materials and methods

### Ethics committee approval and recruitment of subjects

The study was approved by the Institutional Review Board and the Ethics Committee (Ethics Ref. No. LEC 12-15-122) of L. V. Prasad Eye Institute, Hyderabad, India and it adhered to the tenets of Helsinki for research involving human subjects. Three cohorts of individuals were recruited and they included healthy human controls (HC), people with T2DM without DR and people with T2DM and clinically manifest DR. At the time of recruitment, the blood sugar levels of the T2DM and DR individuals were as follows: Fasting Blood Sugar (FBS) level > 120 mg% and Post Prandial Blood sugar (PPBS) level > 200 mg%. In addition the Glycated haemoglobin-A1c (HbA1c) was > 7.0%. All these tests were done by an in house physician using standard clinical protocols. T2DM and DR individuals also had a history of taking anti-diabetic medications (Table 1 and S1 Table). T2DM individuals who had DR lesions in fundus photograph were also confirmed on fundus fluorescein angiography (FFA) and optical coherence tomography (OCT) (Table 1). Table 1 also lists the demographic characteristics of the recruited individuals whose microbiomes have been taken for analysis and the inclusion and exclusion criteria. S1 Table lists the diet and clinical characteristics of the recruited individuals. The population prevalence of DR in India was estimated to be 16.9% for a population of 63,000 [58]. Using the population-proportion method with an 85% confidence level, the sample size derived was 28. Hence 28 patients with DR were recruited in the study. Written informed consent was taken from all the study participants prior to sample collection.

**Table 1:** Demographic characteristics and inclusion and exclusion criteria for the recruitment of Healthy controls (HC), Type 2 Diabetes Mellitus (T2DM) and Diabetic Retinopathy (DR) individuals.

Recruitment	Qualification	HC (n = 30)	T2DM (n = 21)	DR (n = 24)
Inclusion	Age (years)*	Range—38–81	Range—41–69	Range—44–69
		Mean—52.2	Mean—57.5	Mean—54.5
	Gender*	males—17	males—13	males—18
		females -13	females -8	females—6
	Region	Telangana—30	Telangana—20	Telangana– 19
			Andhra Pradesh- 1	Andhra Pradesh—5
	Diet*	Non veg: 23	Non Veg: 20	Non Veg: 20
		Veg: 7	Veg: 1	Veg: 4
	Low sugar diet	None	None	None
	Hypertension	None	11	17
	DM	None	DM: 21	None
			^†^New DM: 13	
			^‡^Known DM: 8	
	DR	None	None	^§^NPDR: 6; ^||^PDR: 18
	Diagnosis	None	History of taking medicines	History of taking medicines
			FBS > 120 mg%	FBS > 120 mg%,
			PPBS > 200mg%	PPBS > 200mg%
			HbA1c > 7.0%	HbA1c > 7.0%
				^#^Fundus photography
	Diabetes Medication^+++^	0	21	24
	Anti-hypertension medication	0	11	17
Exclusion	Prior use of antibiotic, antifungal medicine, prebiotic and probiotic in past 90 days			
	Prior intraocular surgery in past 90 days			
	Intravitreal anti VEGF injection in past 90 days			
	Intravitreal implantable steroid in past 90 days			
	Periocular infection in past 90 days			
	Uncontrolled glaucoma			
	Any form of malignancy			
	Prior gastrointestinal tract surgery			
	Kidney disease, cardiovascular disease, obesity, inflammatory bowel disease, prolonged constipation or diarrhea			

* Indicates p > 0.05.

^†^New-DMs, were individuals who were diagnosed with T2DM recently and are taking anti-diabetic medication for the last 4 to 25 days.

^‡^Known-DMs, were known T2DM individuals and on anti-diabetic medication for the last 1 year.

^§^NPDR- Non-Proliferative Diabetic Retinopathy;

^||^PDR—Proliferative Diabetic Retinopathy.

^#^FFA and OCT were done only in people who had DR lesions in fundus photograph.

FFA—fundus fluorescein angiography; OCT- optical coherence tomography.

^+++^ Metformin or combinations of Metformin and / or Insulin.

### Sample collection, DNA extraction and PCR amplification

The data reported here is part of a large study documenting the changes in the gut microbiome of patients with ocular diseases (bacterial keratitis, fungal keratitis, uveitis, T2DM and DR) compared to HC. Since these studies were undertaken simultaneously, a few of the individuals in the HC cohort were identical. The data of 13 of 30 HC samples were reported in our earlier reports [18, 19, 59]. Fecal samples were collected by the study subjects at home in a sterile container (HiMedia, India) without any storage medium and delivered within 4 hours to LVPEI at room temperature. Samples were frozen at -80°C until further processing.

Genomic DNA was extracted from fecal samples using QIAamp DNA stool minikit (Qiagen, Hilden, North Rhine-Westphalia, Germany) according to the manufacturer’s instructions with few modifications. The collected fecal samples were mixed manually using a sterile spatula (HiMedia, India) until it formed a homogeneous mixture. Then approximately 300 mg of sample was transferred into a 2 ml centrifuge tube and extraction was carried out following the manufacturer’s protocol. For each sample, the extraction was performed in duplicates. At the last step, DNA was eluted with 100 μl of AE buffer provided by Qiagen. Then, equal volume of DNA was taken from each replicate and pooled together for PCR amplification and sequencing. Quality of genomic DNA was checked on 0.8% agarose gel for the presence of a single intact band and quantified using Qubit dsDNA HS Assay kit (Life Tech, India) in Qubit^®^ 2.0 Fluorometer. ITS2, a region of the fungal ribosomal small subunit RNA was amplified with primers ITS3 (5'-GCATCGATGAAGAACGCAGC-3') and ITS4 (5'-TCCTCCGCTTATTGATATGC-3') [19]. PCR reagents were prepared using sterile nuclease free water. The PCR reaction mixture (20 μl) contained 1X PCR buffer, 1.5 mM MgCl_2_, 400 μM deoxyribonucleotide triphosphates, 0.5 μM of each primer, 0.5 U of Taq DNA polymerase and template DNA (~ 50 ng). The thermal profile for amplification comprised an initial denaturation of 10 min at 95°C, followed by 39 cycles of denaturation at 95°C for 1 min, annealing at 56°C for 1 min and elongation at 72°C for 1 min and a final elongation of 10 min at 72°C. PCR was negative for the reagents used for DNA extraction and for the PCR reaction mix containing all the PCR components, without template DNA. Sequencing of these PCR negative reactions did not yield any fungal reads.

### Illumina library preparation and amplicon sequencing

The amplicon libraries were prepared using Nextera XT Index Kit (Illumina Inc., San Diego, California, USA) as per the ITS Metagenomic Sequencing Library preparation protocol (Part # 15044223 Rev. B). The amplicons with the Illumina adaptors were amplified using i5 and i7 primers that add multiplexing index sequences as well as common adapters required for cluster generation (P5 and P7). The amplicon libraries were purified by 1X AMpureXP beads, checked on Agilent DNA 1000 chip on Bioanalyzer 2100 and quantified by Qubit Fluorometer 2.0 using Qubit dsDNA HS Assay kit (Life Technologies, India). After obtaining the Qubit concentration for the library and the mean peak size from Bioanalyser profile, the library was spiked with 50% PhiX control v3 (FC-110-3001) as described in the Illumina procedure and loaded onto illumina NGS platform at an appropriate concentration (10–20 pM) for cluster generation and sequencing. The libraries were sequenced at Xcelris Genomics Pvt. Ltd. (Ahmedabad, India), using Illumina HiSeq 2 X 250 base pair chemistry. Sequencing of PCR negative reactions did not yield any fungal reads.

### Taxonomy assignment of sequenced reads

Paired-end reads of each sample were assembled through FLASH software [60]. Low quality (mean Phred score < 25) and chimeric sequences were removed with Prinseq-lite [61] and Usearch61 [62] respectively. The retained high quality (HQ) reads were used for operational taxonomic unit (OTU) picking with an ‘open reference OTU picking’ method in the Quantitative Insights into Microbial Ecology (QIIME) pipeline [63] using UNITE OTUs (ITS) version 8.2 [64] clustered at 97% sequence similarity. Taxonomic assignments of denovo-OTUs were attained using Wang Classifier [65, 66] with a bootstrap threshold of 80%. OTUs containing < 0.001% of the total number of reads assigned to OTUs (sparse OTUS) were excluded from further analysis.

Batch effect in the mycobiomes was removed using the ComBat function in the package SVA [67] to overcome variations between samples of the same cohort since they were analysed at different points of time using the same protocol and NGS platform. Extraction of genomic DNA and sequencing were done in two different batches since the availability of the samples was dependent on the recruitment of subjects. Batch I included 13 HC (HC005-HC028), 10 T2DM (T2DM001-T2DM012) and 6 DR (DR002-DR013) samples and batch II included 17 HC (HC0037-HC053), 11 T2DM (T2DM013-T2DM025) and 18 DR (DR014-DR031) samples. Samples in both the batches were analysed together up to OTU picking and taxonomy assignment. Consequently, the abundance table was split on the basis of cohorts and batch effect correction was applied to each cohort separately. At the end, the batch effect corrected OTUs abundance was merged and used for all further analysis.

### Diversity analyses of the mycobiomes

Rarefaction curves and Alpha diversity indices (Shannon diversity, Simpson index, number of observed OTUs, and Chao1 index) were plotted using R-Vegan 2.4–2 package (http://vegan.r-forge.r-project.org/). Significant differences in Alpha diversity indices between the groups were determined by t-test.

### Identification of differentially abundant taxonomic groups

Kruskal-Wallis and Wilcoxon signed rank tests were performed to identify the differentially abundant taxonomic groups [Benjamini Hochberg (BH) corrected *P* < 0.05] between HC, T2DM and DR samples (at the phylum and genus level) in the mycobiomes. Differences at the genera level were also visualized through non-metric multidimensional scaling (NMDS) plots using Bray-Curtis dissimilarity. NMDS plots were generated using the discriminatory genera between the cohorts. The linear discriminant analysis effect size method (https://huttenhower.sph.harvard.edu/galaxy) was used to observe the mycobiome features significantly associated with T2DM and DR at various taxonomic levels.

### Interaction networks between fungal genera in the mycobiomes

Pair-wise correlations between abundances of different fungal genera which were obtained using Spearman correlation coefficient (r) were used to generate separate interaction networks with the help of CoNet [68] in Cytoscape [69].

### Correlation of fungal genera in HC, T2DM and DR mycobiomes

Correlation analysis of fungal microbiomes was performed with genera having a median abundance of > 0.5 by Spearman’s rank correlation using Corrplot package in R.

## Results

### Analysis of the gut mycobiomes

From the 83 fecal samples (30 HC, 25 T2DM and 28 DR), ITS2 mycobiomes were generated from 79 samples (30 HC, 23 T2DM and 26 DR). The remaining 4 samples did not yield ITS2 amplicons. Mycobiomes, in which 80–85% of the reads were assigned as unclassified or dominated by only one genus were also excluded from the study. Eventually 30 HC, 21 T2DM and 24 DR mycobiomes were analysed. Several confounding factors could influence the gut mycobiome of individuals. Conscious of this fact, 30 HC, 21 T2DM and 24 DR individuals in the 3 cohorts were age, gender, region, diet and ethnicity matched (*P* > 0.05). The individuals were either vegetarians or non-vegetarians and were matched across the cohorts (*P* = 0.203). This would help to ascertain that changes observed in the 3 cohorts are related to their health status and not influenced by any confounding factor.

### Sequencing coverage and diversity indices of the gut fungal mycobiomes of HC, T2DM and DR individuals

The 30 HC, 21 T2DM and 24 DR mycobiomes generated 17.27, 10.67 and 8.56 million high quality (HQ) reads (after removal of chimeric reads and reads with < 25 mean Phred score) respectively. No significant difference was observed in the number of HQ reads among the three cohorts (*P* = 0.34). Further, the average number of HQ reads per mycobiome was 0.58, 0.51 and 0.36 million in HC, T2DM and DR respectively. We noted that the majority of the HQ reads (89.47 to 99.43%) were assigned to an OTU. In total, 977 OTUs were identified in the three cohorts and it included 33 reference and 944 denovo OTUs (S2 Table). Rarefaction curves of the 75 mycobiomes consistently attained the plateau phase indicating that the sequencing depth and coverage were sufficient to cover the total fungal diversity in the mycobiomes (S1 Fig).

**Fig 1:**
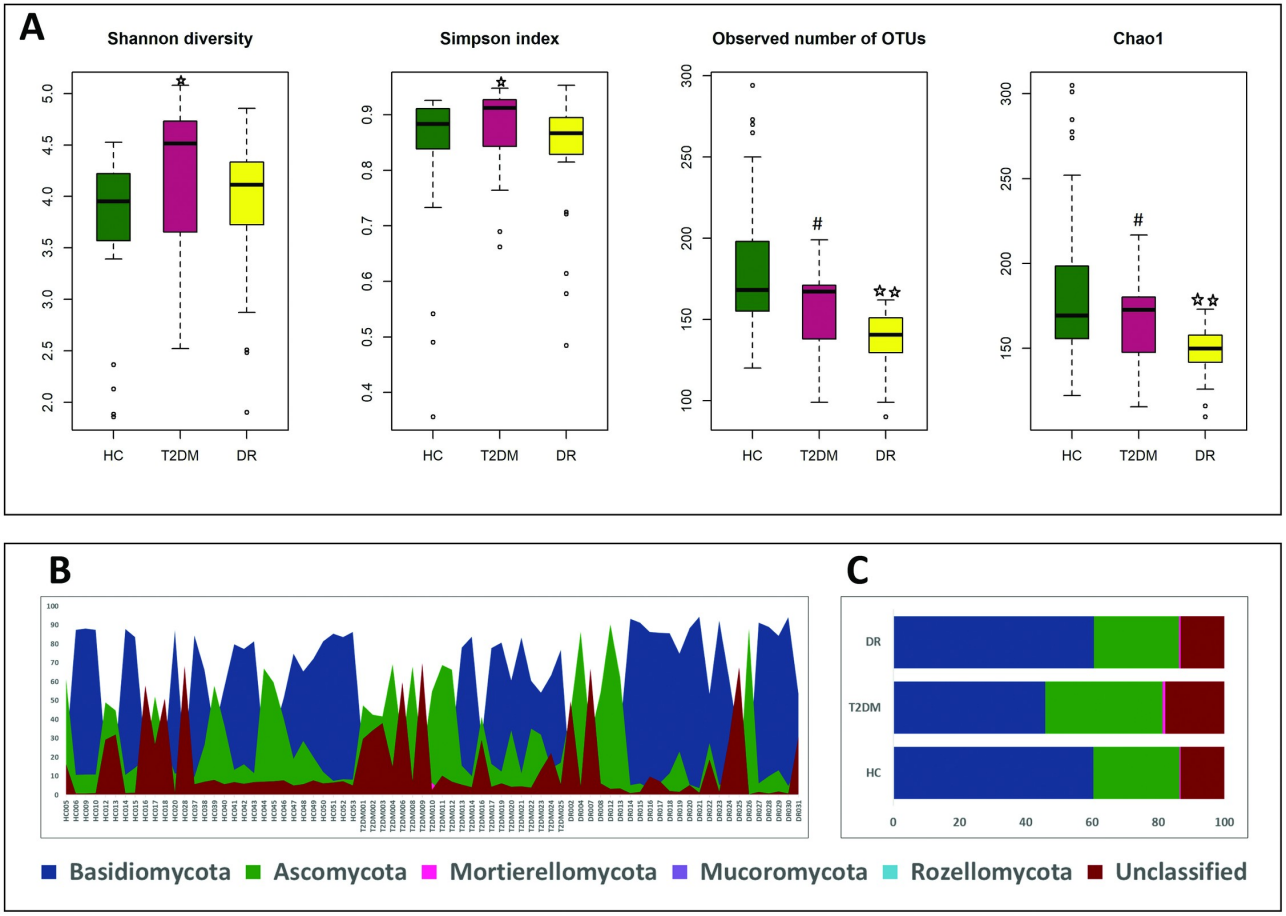
Fungal diversity in the gut mycobiomes of healthy controls (HC, n = 30), Type 2 Diabetes Mellitus (T2DM, n = 21) and Diabetic Retinopathy (DR, n = 24) individuals. **(A)** Of the four Alpha diversity indices, Shannon and Simpson indices were statistically significant between HC and T2DM (indicated by ★) and the observed number of OTUs and Chao1 index were statistically significant between HC and DR (indicated by ★★) and T2DM and DR (indicated by **#**) (*P* = < 0.05). **(B)** Abundance and **(C)** mean abundance (%) of different fungal phyla.

Observed number of OTUs, Shannon and Chao1 indices were significantly different across all the three cohorts (HC, T2DM and DR) using Kruskal-Wallis test. Student’s t-test also indicated that Shannon and Simpson indices were statistically significant between HC and T2DM, whereas observed number of OTUs and Chao1 index were statistically significant between HC and DR and T2DM and DR (Fig 1A). Rarefaction was used to adjust the difference in the library sizes and then the Alpha diversity was calculated with 50,000 reads per sample. This yielded the same results (S2 Fig).

### Analysis of the gut mycobiomes of HC, T2DM and DR patients at the phylum level

The number of OTUs across the individual mycobiomes that were taxonomically assigned to a phylum varied from 68 (T2DM014) to 210 (HC018) (S3 Table). Fungal phyla (Basidiomycota and Ascomycota) were consistently detected in HC, T2DM and DR mycobiomes. In abundance, Basidiomycota was the most dominant phylum (mean abundance 45.83 to 60.64%) followed by Ascomycota (mean abundance 25.68 to 35.49%) and Mortierellomycota which were present in majority of the mycoobiomes (mean abundance 0.3 to 0.72%). Mucoromycota was also present in all the HC microbiomes (mean abundance 0 to 0.02%) but only in a very few T2DM and DR mycobiomes. Rozellomycota represents a minor phylum present only in some of the microbiomes of HC and T2DM mycobiomes (S4 Table, Table 2, Fig 1B and 1C). It was also observed that the abundance of Mucoromycota was significantly different between HC and T2DM, and between HC and DR (*P* < 0.05).

**Table 2:** Mean abundance (%) of fungal phyla in the gut mycobiomes of healthy controls (HC, n = 30), Type 2 Diabetes Mellitus (T2DM, n = 21) and Diabetic Retinopathy (DR, n = 24) patients.

S. No.	Phylum	HC			T2DM			DR			Wilcoxon test P value (BH corrected)*		
		Abundance		Present Out of 30 samples	Abundance		Present Out of 21 samples	Abundance		Present Out of 24 samples	HC vs. T2DM	HC vs. DR	T2DM vs. DR
		Mean	Range		Mean	Range		Mean	Range				
1	Basidiomycota	60.49	18.32–88.07	30	45.83	10.62–83.79	21	60.64	1.91–94.41	24	0.098	0.373	0.102
2	Ascomycota	25.91	7.45–67.07	30	35.49	10.03–69.25	21	25.68	3.24–90.42	24	0.098	0.224	0.051
3	Mortierellomycota	0.3	0–0.94	27	0.72	0–6.62	15	0.47	0–2.88	20	0.825	0.373	0.775
4	Mucoromycota	0.02	0–0.15	30	0	0–0.01	3	0	0–0.04	9	**0**	**0**	0.102
5	Rozellomycota	0	0–0.02	6	0.03	0–0.61	1	0	0–0	0	0.232	0.069	0.367
6	Unclassified	13.28	0.82–68.31	30	17.92	2.79–69.96	21	13.21	0.26–67.73	24	0.571	0.304	0.051

*P value of 0 indicates ≤ 0.0001.

### Differentially abundant fungal genera in the gut fungal mycobiomes of HC, T2DM and DR patients

The number of OTUs across the individual mycobiomes taxonomically assigned to a genus varied from 53 (T2DM014) to 156 (HC028) (S5 Table). In total, 107 genera were present in HC mycobiomes, with 29 genera present in all the HC mycobiomes and 78 genera in 3% to 97% of the HC mycobiomes. In T2DM mycobiomes, 120 genera were detected with 11 genera shared between all the T2DM mycobiomes and the other 109 genera were present in 5% to 90% of the T2DM mycobiomes. In DR mycobiomes, a total of 115 genera were detected with 8 genera shared between the DR mycobiomes. The remaining 107 genera were present in 4% to 92% of the DR mycobiomes (S6 Table).

**Fig 2:**
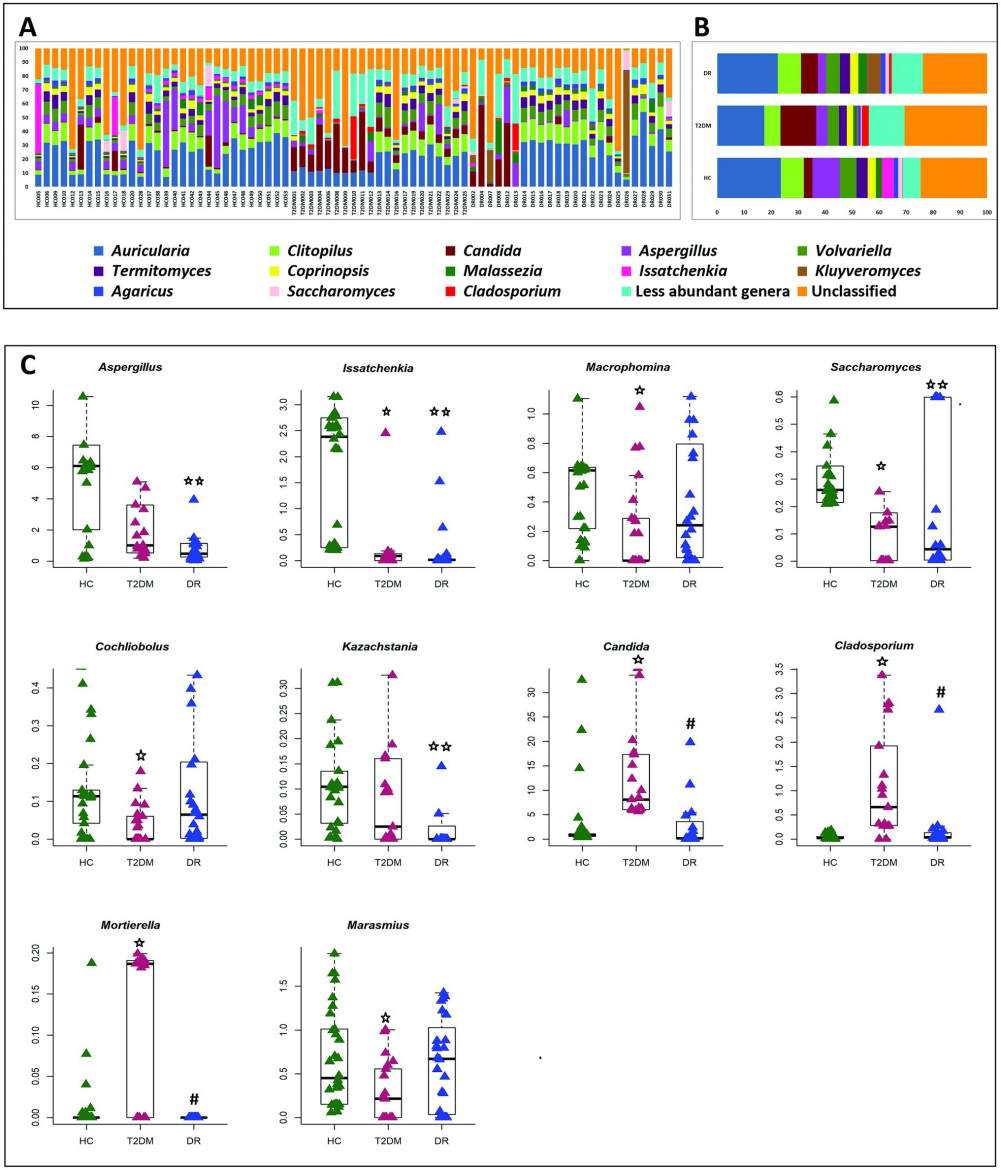
Diversity of different fungal genera in the gut mycobiomes of healthy controls (HC, n = 30), Type 2 Diabetes Mellitus (T2DM, n = 21) and Diabetic Retinopathy (DR, n = 24) patients. **(A)** Abundance and **(B)** mean abundance (%) of different fungal genera. Genera with < 1% mean abundance were categorized as ‘‘less abundant genera”. **(C)** Fungal genera exhibiting significant (Wilcoxon test, BH corrected p < 0.05) differential abundance in the gut mycobiomes HC, T2DM and DR patients. Median abundances (horizontal line) and inter-quartile ranges are indicated in the plots. Statistically significantly abundant genera between HC and T2DM are indicated by ★, between HC and DR are indicated by ★★ and between T2DM and DR are indicated by **#**.

A total of 150 genera were identified in the 75 gut mycobiomes of HC, T2DM and DR patients (S6 Table). The diversity between the cohorts was similar but not identical (Fig 2A and 2B). HC shared 82 genera with T2DM and 84 genera with DR; HC had 21 unique genera. Between T2DM and DR, 106 genera were shared (S7 Table). Despite these diversity similarities, comparison of the abundance of fungal genera between the three cohorts indicated the following: (1) abundance of 21 genera in T2DM and 18 in DR were reduced; (2) 5 genera were significantly enriched only in T2DM patients compared to HC (Tables 3 and 4); and (3) 6 genera were reduced in DR compared to T2DM patients (Table 5). Fig 2C depicts the relative abundance of some of the discriminating genera in HC, T2DM and DR.

**Table 3:** Fungal genera exhibiting significant differential abundance (BH corrected *P* ≤0.05) between the gut mycobiomes of Healthy controls (HC, n = 30) and Diabetes Mellitus (T2DM, n = 21) individuals.

S. No.	Genus	Median Abundance (%)*		Wilcoxon test—*P* value (BH corrected *P* value ≤ 0.05)†	Pathogenicity
		HC	T2DM		
**Genera decreased in T2DM**					
1	*Issatchenkia*	2.387	0.093	0	Plant/Human pathogen
2	*Macrophomina*	0.616	0	0	Plant pathogen
3	*Marasmius*	0.451	0.215	0	Commensal
4	*Gymnopilus*	0.308	0.123	0.043	Commensal
5	*Saccharomyces*	0.261	0.127	0	Non pathogen
6	*Trichoderma*	0.134	0.05	0.043	Plant pathogen
7	*Cochliobolus*	0.114	0	0	Plant pathogen
8	*Psathyrella*	0.102	0.031	0.043	Commensal
9	*Clavispora*	0.027	0.001	0	Not known
10	*Didymella*	0.011	0.002	0	Plant pathogen
11	*Ganoderma*	0.007	0	0.043	Plant pathogen
12	*Rhizopus*	0.006	0	0	Plant/Human pathogen
13	*Mycosphaerella*	0.005	0.003	0.043	Plant pathogen
14	*Wickerhamomyces*	0.005	0	0	Antimicrobial
15	*Gliocladiopsis*	0.004	0	0	Plant pathogen
16	*Cuphophyllus*	0.002	0	0	Commensal
17	*Cylindrocladiella*	0.002	0	0	Plant pathogen
18	*Backusella*	0.001	0	0	Not known
19	*Corynespora*	0.001	0	0	Plant pathogen
20	*Vishniacozyma*	0.001	0	0.043	Antimicrobial
21	*Volutella*	0.001	0	0.043	Plant pathogen
**Genera increased in T2DM**					
1	*Candida*	0.878	8.119	0	Human pathogen
2	*Cladosporium*	0.023	0.658	0	Plant pathogen
3	*Kodamaea*	0.001	0.014	0.043	Human pathogen
4	*Meyerozyma*	0.001	0.067	0	Human pathogen
5	*Mortierella*	0	0.187	0	Soil fungi

*Differentially abundant genera having a median abundance ≥ 0.001% in at least one group of samples are listed.

^†^*P* value of 0 indicates ≤ 0.0001.

**Table 4:** Fungal genera exhibiting significant differential abundance (BH corrected *P* ≤ 0.05) between the gut mycobiomes of Healthy controls (HC, n = 30) and Diabetic Retinopathy (DR, n = 24) individuals.

S. No.	Genus	Median Abundance (%)*		Wilcoxon test—P value (BH corrected P value ≤ 0.05)†	Pathogenicity
		HC	DR		
Genera decreased in DR					
1	*Aspergillus*	6.116	0.48	0	Human pathogen
2	*Issatchenkia*	2.387	0.016	0	Plant/Human pathogen
3	*Saccharomyces*	0.261	0.044	0.045	Non pathogen
4	*Kazachstania*	0.104	0	0	Not known
5	*Didymella*	0.011	0	0	Plant pathogen
6	*Diutina*	0.01	0	0	Human pathogen
7	*Rhizopus*	0.006	0	0	Plant/Human pathogen
8	*Mycosphaerella*	0.005	0.001	0.045	Plant pathogen
9	*Wickerhamomyces*	0.005	0	0	Antimicrobial
10	*Gliocladiopsis*	0.004	0	0	Plant pathogen
11	*Oliveonia*	0.004	0	0.045	Not known
12	*Cylindrocladiella*	0.002	0	0	Plant pathogen
13	*Pseudogymnoascus*	0.002	0	0	Animal pathogen
14	*Backusella*	0.001	0	0	Not known
15	*Cladorrhinum*	0.001	0	0	Human pathogen
16	*Corynespora*	0.001	0	0	Plant pathogen
17	*Vishniacozyma*	0.001	0	0	Antimicrobial
18	*Volutella*	0.001	0	0.045	Plant pathogen

*Differentially abundant genera having a median abundance ≥ 0.001% in at least one group of samples are listed.

^†^*P* value of 0 indicates ≤ 0.0001.

**Table 5:** Fungal genera exhibiting significant differential abundance (BH corrected *P* ≤ 0.05) between the gut mycobiomes of Diabetes Mellitus (T2DM, n = 21) and Diabetic Retinopathy (T2DM, n = 24) individuals.

S. No.	Genus	Median Abundance (%)*		Wilcoxon test—P value (BH corrected P value ≤ 0.05)†	Pathogenicity
		T2DM	DR		
Genera decreased in DR					
1	*Candida*	8.119	0.168	0	Human pathogen
2	*Cladosporium*	0.658	0.041	0	Plant pathogen
3	*Mortierella*	0.187	0	0	Soil fungi
4	*Meyerozyma*	0.067	0	0	Human pathogen
5	*Kodamaea*	0.014	0	0	Human pathogen
6	*Didymella*	0.002	0	0	Plant pathogen

*Differentially abundant genera having a median abundance ≥ 0.001% in at least one group of samples are listed.

^†^*P* value of 0 indicates ≤ 0.0001.

We also categorised T2DM patients into two subgroups namely new-T2DM (recently diagnosed as T2DM and are on anti-diabetes medication for < 1 month, n = 13) and known-T2DM (patients with T2DM and taking anti-diabetes medication for at least 1 year, n = 8). We observed that out of 120 genera, only one genus (*Candida*) was significantly enriched in new T2DM and 8 genera (*Agaricus*, *Chlorophyllum*, *Coprinopsis*, *Leucoagaricus*, *Termitomyces*, *Trametes*, *Trichoderma* and *Volvariella*) were significantly reduced in new T2DM through Wilcoxon test.

DR was also divided into two subgroups namely ‘PDR’ (Proliferative Diabetic retinopathy, n = 18) and ‘NPDR’ (Non-Proliferative Diabetic Retinopathy, n = 6). Wilcoxon test did not identify any discriminatory genera between the DR subgroups implying that the mycobiomes were similar and degree of DR did not influence the results.

**Fig 3:**
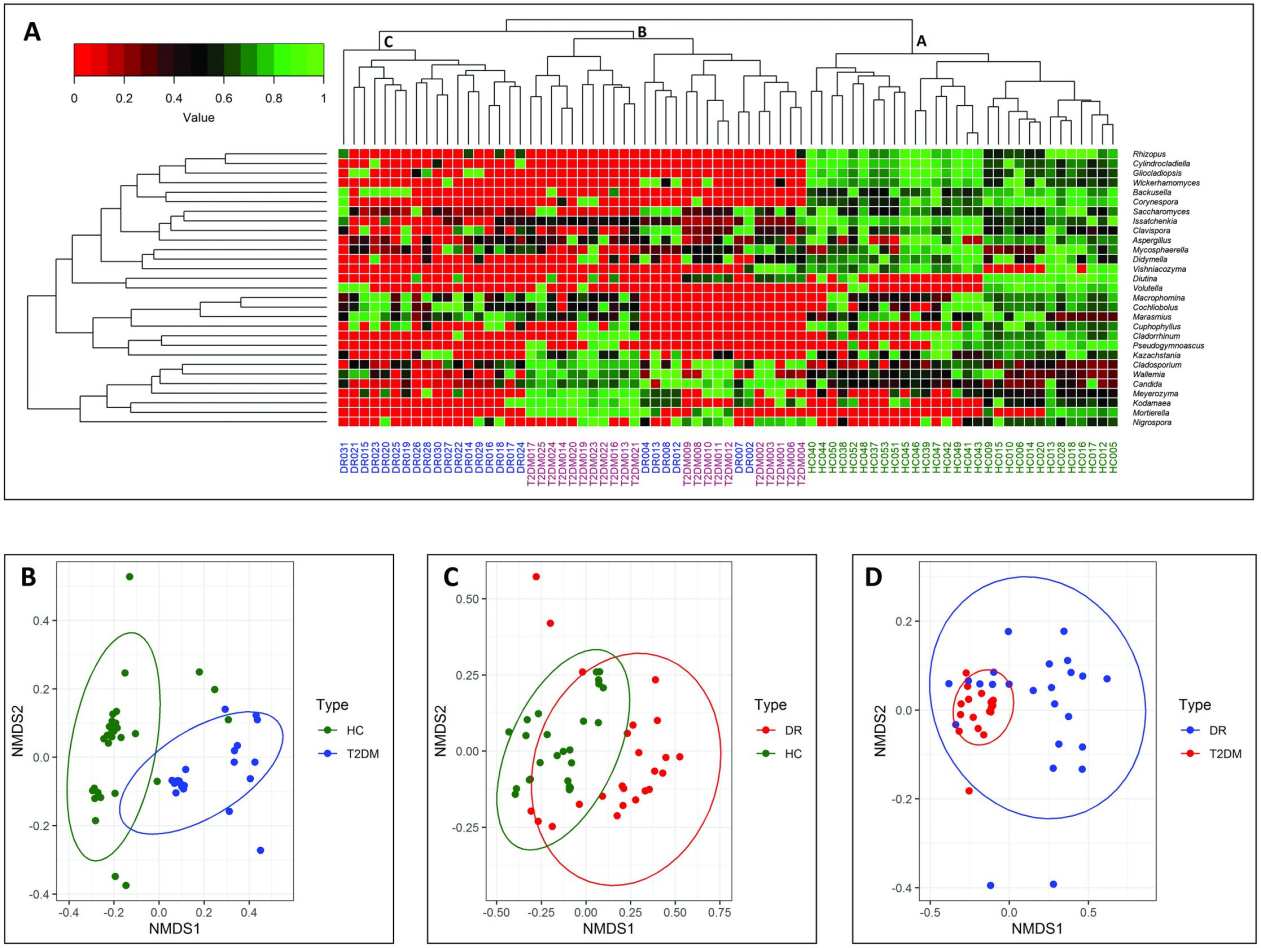
Gut fungal mycobiomes differ significantly across healthy controls (HC, n = 30), Type 2 Diabetes Mellitus (T2DM, n = 21) and Diabetic Retinopathy (DR, n = 24) patients. **(A)** Two dimensional heat map showing rank normalized abundances (scaled between 0 and 1) of 29 differentially abundant fungal genera determined by Wilcoxon test. Discriminating genera have been arranged along the two dimensions (axes) based on hierarchical clustering. **(B)** Beta diversity analysis using NMDS plots based on Bray-Curtis dissimilarity of discriminating genera in the gut microbiomes of HC and T2DM, **(C)** HC and DR and **(D)** T2DM and DR. The fecal mycobiomes appeared to vary significantly (PERMANOVA, *P* = 0.001).

Two-dimensional heat map analysis with 29 discriminating fungal genera indicated that mycobiomes of HC and diseased individuals (T2DM and DR) formed three distinct clusters namely A, B and C with three sub-clades in each. All the mycobiomes of HC and T2DM grouped together in clades A and B respectively, whereas majority of the DR mycobiomes (18 of 24) grouped into sub-clade C and the remaining 6 mycobiomes were interspersed in the T2DM clade B (Fig 3A). Beta diversity analysis using NMDS plots based on Bray-Curtis dissimilarity of discriminating genera also clearly segregated the gut mycobiomes of HC and T2DM (*P* = 0.001), HC and DR (*P* = 0.001) and T2DM and DR (*P* = 0.001) (Fig 3B–3D). The *P*-value was calculated using PERMANOVA. Linear discriminant analysis effect size method (LEfSE) with OTU abundance showed the mycobiome features of HC, T2DM and DR at various taxonomic levels (S3 Fig).

### Interactions between the fungal genera in the gut fungal mycobiomes of HC, T2DM and DR patients

The three interaction networks (HC, T2DM and DR) generated based on pair-wise correlations between abundances of different fungal genera (S4 Fig) depicted a single, well-connected large network along with several disjointed smaller networks (S4 Fig). Several ‘hub’ genera (exhibiting > 10 positive or negative or both interactions) were identified in HC (n = 20), T2DM (n = 15) and DR (n = 15) mycobiomes. In HC, 14 hub genera (*Pichia*, *Thyrostroma*, *Ciliophora*, *Coprinus*, *Preussia*, *Darksidea*, *Cistella*, *Pezoloma*, *Paraphoma*, *Gibberella*, *Leptosphaeria*, *Comoclathris*, *Articulospora* and *Mortierella*) were unique and were not seen in T2DM and DR mycobiomes. Three hub genera (*Agaricus*, *Termitomyces* and *Volvariella*) were common to HC, T2DM and DR cohorts and only one hub genus, *Trichoderma*, was shared only between HC and T2DM cohorts. Between HC and DR, *Auricularia* and *Coprinopsis* were shared. In T2DM, 5 genera (*Plectosphaerella*, *Candida*, *Xeromyces*, *Leucoagaricus* and *Coprinellus*) formed unique hubs whereas in DR, 4 genera (*Rhodosporidiobolus*, *Cutaneotrichosporon*, *Blastobotrys* and *Saccharomyces*) were the unique hubs. Nine hub genera (*Agaricus*, *Termitomyces Volvariella*, *Hortaea*, *Chlorophyllum*, *Marasmius*, *Clitopilus*, *Gymnopilus* and *Psathyrella*) were common to T2DM and DR cohorts. In general, the HC, T2DM and DR interaction networks were different.

### Correlation of fungal genera in HC, T2DM and DR

**Fig 4:**
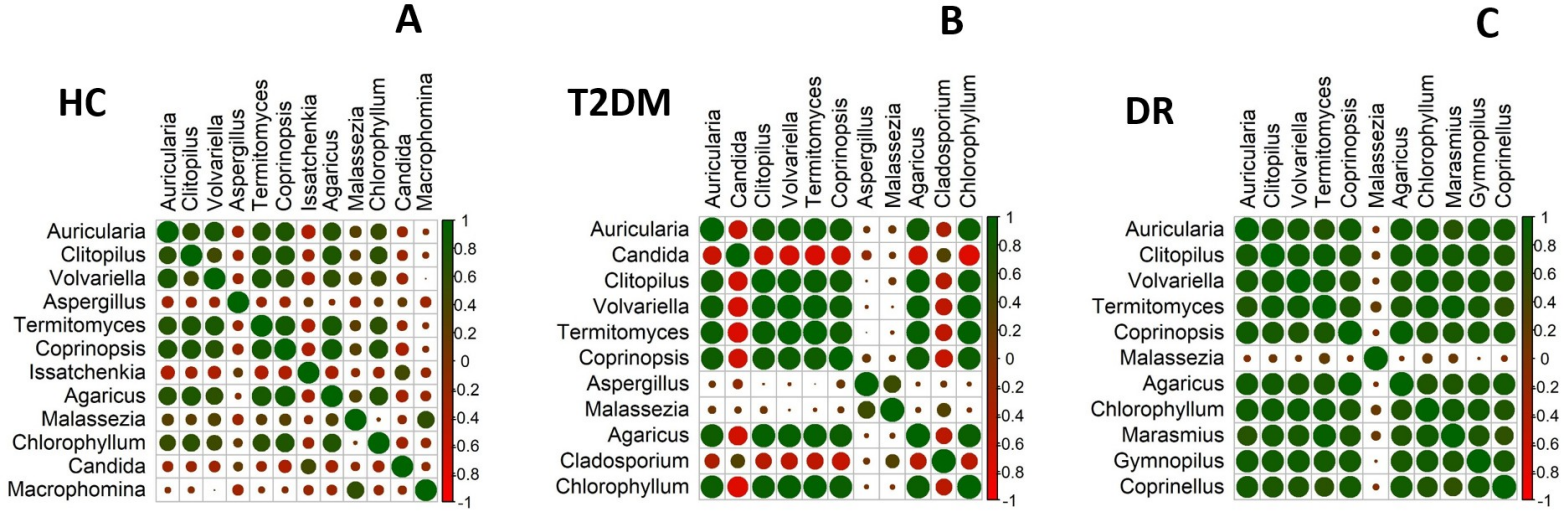
Spearman correlation. Correlation analysis of fungal genera having a median abundance of > 0.5% in the gut mycobiomes of **(A)** healthy controls (HC, n = 30), **(B)** Type 2 Diabetes Mellitus (T2DM, n = 21) and **(C)** Diabetic Retinopathy (DR, n = 24) patients.

Correlation analysis (Spearman correlation) of fungal genera with median abundance of > 0.5% (Fig 4A–4C) indicated that 7 genera (*Auricularia*, *Clitopilus*, *Volvariella*, *Termitomyces*, *Coprinopsis*, *Agaricus* and *Chlorophyllum*) correlated positively with most of the genera in the gut mycobiomes of HC and T2DM whereas in DR mycobiomes, in addition to the above 7 genera, *Marasmius*, *Gymnopilus* and *Coprinellus* also showed positive correlation. *Aspergillus*, *Issatchenkia*, *Malassezia*, *Candida* and *Macrophomina* displayed an overall high negative correlation with all other genera in HC, whereas in T2DM *Candida*, *Aspergillus*, *Malassezia* and *Cladosporium* and in DR only *Malassezia* exhibited a negative correlation. Among the three cohorts, the genera of HC showed more negative correlations and the genera in DR showed more positive interactions.

## Discussion

### Gut mycobiome of HC individuals

The most comprehensive study on the gut mycobiomes in healthy individuals (over 100 volunteers) from Texas, USA, indicated that 15 fungal genera (*Saccharomyces*, *Malassezia*, *Candida*, *Cyberlindnera*, *Penicillium*, *Cladosporium*, *Fungi spp*., *Aspergillus*, *Agaricus*, *Fusarium*, *Pichia*, *Debaryomyces*, *Galactomyces*, *Alternaria* and *Clavispora*) were most abundant [70]. In the present study 12 of these 15 fungi were detected in the healthy mycobiomes and three genera (*Cyberlindnera*, *Debaryomyces* and *Galactomyces*) were not detected in any of the healthy mycobiomes. The 12 genera shared in the healthy controls from India and the USA may imply that such genera are the characteristics of the gut mycobiome. In addition to the above genera, we also detected 28 other genera (*Auricularia*, *Clitopilus*, *Volvariella*, *Termitomyces*, *Coprinopsis*, *Issatchenkia*, *Chlorophyllum*, *Macrophomina*, *Marasmius*, *Coprinellus*, *Gymnopilus*, *Blumeria*, *Psathyrella*, *Lasiodiplodia* etc.,) which were present in 28 of the 30 (>90%) healthy Indian mycobiomes (S7 Table). Discrepancy in identification of a genus or genera could also be due to the differences in methodologies or in the cohorts itself as observed in volunteers from Houston and Pennsylvania for the genus *Malassezia* [70, 71]. Our study also confirms that the gut mycobiomes are highly variable between individuals [70, 72]. The fungi identified in this study (*Saccharomyces*, *Malassezia*, *Candida*, *Penicillium*, *Cladosporium*, *Aspergillus* etc.) have also been reported by the conventional culture based methods [39, 42, 49, 73]. Nash et al. [70] reported that strongest positive correlation is exhibited between *Sarocladium* and *Fusarium*, and strongest negative correlation is exhibited between *Candida* and *Saccharomyces* in human mycobiomes. This is in variance to our observations. In our study 7 genera (*Auricularia*, *Volvariella*, *Termitomyces*, *Coprinopsis*, *Agaricus*, *Clitopilus* and *Chlorophyllum*) correlated positively and *Aspergillus*, *Issatchenkia*, *Malassezia*, *Candida* and *Macrophomina* correlated negatively with most other genera in the mycobiomes of human control.

### Gut mycobiome changes in people with T2DM

Changes in the fungal microbiota in people with T2DM using either conventional culture based methods or quantitative real time PCR [40, 54, 55] demonstrated differences in the gut mycobiota in T1DM and/or T2DM subjects with an increase in *Candida* species. This observation was also confirmed by gut mycobiome analysis in T2DM patients [74]. Our results indicated that Mucoromycota was the only phylum that showed significant reduction in abundance in T2DM compared to the control mycobiomes. This phylum was detected only in 14% of the T2DM mycobiomes though it was detected in all the mycobiomes of the control (Table 2, Fig 1B and 1C). The median abundance of *Candida* along with 4 other genera (*Cladosporium*, *Kodamaea*, *Meyerozyma* and *Mortierella*) were increased in people with T2DM (Table 3). Many of these genera which increased in abundance in T2DM are known pathogens that includes 3 human pathogens (*Candida*, *Kodamaea* and *Meyerozyma*) [75], one plant pathogen (*Cladosporium*), and one soil fungi with anti-microbial properties (*Mortierella*) [76]. Such a preponderance of pathogenic fungi, may exert a pro-inflammatory response and thus may support T2DM which is an inflammatory disease. Concomitantly, decreased abundance of 21 genera in people with T2DM was observed and these included plant/human pathogens (12 genera), commensal fungi (4 genera), non-pathogens (1 genus), genera with antimicrobial properties (2 genera) and 2 genera with no function (Table 3). All commensal fungi are observed in the gut mycobiomes in healthy controls and these fungi may not have a specific role in the mammalian gastrointestinal tract [31, 42, 46, 77, 78]. Thus, our study demonstrates dysbiosis (at the diversity, abundance and functional level) in the mycobiomes in people with T2DM compared to healthy controls and confirms similar earlier observations [40, 54, 55].

### Gut mycobiome changes in people with DR

This is the first study that has attempted to analyse the gut mycobiomes of people with DR. The results indicated that the Mucoromycota was the only phylum that showed significant reduction in abundance in DR compared to the control mycobiomes (Table 2). Further, eighteen genera decreased in abundance in DR compared to HC. Interestingly, 12 of the 18 genera that decreased in DR were also decreased in T2DM implying that these genera are not important for T2DM and DR (compare Tables 3 and 4) but important for HC mycobiomes. But, it is surprising that several of these genera that decreased (8 out of 12) were plant/human pathogen though the remaining four genera that decreased were either a non-pathogen (1 genus), with anti-microbial properties (2 genera) and for 1 genus the function was not known (Table 4). This implies that the genera that were decreased (n = 6) exclusively in DR may have a specific role in DR. Four of these genera that were decreased (*Aspergillus*, *Diutina*, *Pseudogymnoascus* and *Cladorrhinum*) were animal or human pathogens; these may have a pro-inflammatory effect. The functions of the remaining 2 genera (*Kazachstania* and *Oliveonia*) is not known and may support DR (Table 4). In the absence of specific functional inputs on other fungi it is difficult to predict their specific role in DR. Further, none of the genera in DR showed any significant increase in abundance compared to T2DM or HC mycobiomes.

### Gut mycobiome changes in people with T2DM and DR

Significant differences in abundance were observed in the mycobiomes of T2DM and DR only at the genera level (Table 5). None of the genera increased in abundance in DR compared to T2DM, but 6 genera decreased in abundance in DR compared to T2DM mycobiomes (Table 5). The genera that decreased included human pathogens (*Candida*, *Meyerozyma* and *Kodamaea* n = 3), plant pathogens (*Cladosporium* and *Didymella* n = 2) and a soil fungus (*Mortierella*, n = 1). But the relative abundances of these fungi were ≤0.168%. It is difficult to predict how this community would influence DR except to predict that the predominating pathogens may have an inflammatory action.

Chronic inflammation is a prerequisite to the onset of DR and this may be mediated by the gut mycobiota. For instance, it has been demonstrated that fungi like *C*. *albicans* and *Aspergillus fumigatus* trigger *in vivo* inflammatory responses [79]. In the present study, *Candida*, *Meyerozyma* and *Kodamaea* increased in T2DM (Table 3), which could have an inflammatory role. Simultaneously, it was also observed that in DR, 6 genera were decreased which included *Aspergillus*, *Diutina*, *Pseudogymnoascus* and *Cladorrhinum* which were animal or human pathogens with possible pro-inflammatory effects. The decrease in abundance implies that these fungi do not support DR but may be required for the T2DM status of the patient.

### Relevance of the gut mycobiome changes in T2DM and DR patients

Dysregulation in the balance between pro- and anti-inflammatory signaling may significantly worsen diseases [80–84]. We anticipated two distinct differences in the mycobiomes between the normal (healthy control) and the diseased states (T2DM and DR). In the healthy controls, there would be an increase in commensal bacteria which may not cause inflammation and decrease in plant and animal pathogens which could support inflammatory conditions (Tables 3 and 4). In contrast in the diseased state (T2DM and DR), there would be a decrease in the abundance of commensal bacteria and concomitant increase in human and plant pathogens that could cause inflammation (Tables 3 and 4). Further between T2DM and DR, specific changes were not anticipated (Table 5). A clear cut trend as anticipated above was not obvious in T2DM and DR mycobiomes. But, overall we did observe increase or decrease in pathogens and decrease in commensals in T2DM mycobiomes whereas in DR only decrease in pathogens was observed (Tables 3–5). These observations of increase in pathogenic fungi in T2DM are also similar to ones with allergic asthma [85] implying that such changes may be common to several diseases mediated by mast cells and aggravate allergic inflammation [86]. The only studies available on ocular diseases include gut mycobiome changes in UVT [59], BK [18] and FK [19]. When we compared the BK and UVT mycobiomes, 18 identical fungi were identified either at the genera or higher level exhibited a decrease in abundance and included fungi that were beneficial to HC due to their anti-inflammatory or anti-pathogenic effects [18, 59]. BK gut mycobiome [18] also showed increase in *Saccharomyces*, which is an opportunistic pathogen, as in UVT mycobiomes [59]. However in the FK mycobiomes, the overall abundance of all the apparently ‘discriminatory’ OTUs were very low (< 0.001%) and were not indicative of any significant dysbiosis [19] and were thus not compared. When BK and UVT mycobiomes were compared with the discriminating genera in DR mycobiomes, it was observed that *Aspergillus* was the only genus that was shared between the mycobiomes of BK, UVT and DR, *Rhizopus* was a discriminatory genus in both BK and DR, whereas *Issatchenkia* was a common discriminatory genus in Uveitis and DR mycobiomes. Thus it would appear that changes in microbiota (at the taxonomic level) may not be common across all diseases, but at the functional level, changes could be observed with respect to increase/decrease in anti- or pro-inflammatory, commensal, probiotic microbes etc.

### Other distinct changes in the gut mycobiomes of healthy controls, and people with T2DM and DR

To our knowledge, till date only one report from India indicated dysbiotic changes at the phyla and genera level in the mycobiomes of people with DM compared to healthy controls [74]. They demonstrated disease-state specific separation. Our study confirms this observation; we noted a clear separation by heatmap analysis. In addition, we demonstrated dysbiosis, at the phyla and genera level, in the gut mycobiomes of DR versus healthy controls and DR versus T2DM. We also demonstrated disease-state specific separation by heatmap and Beta diversity analysis using NMDS plots which segregated the gut mycobiomes of HC and T2DM, HC and DR and T2DM and DR mycobiomes (Fig 3). LEfSE analysis also confirmed differences in the mycobiome features in HC, T2DM and DR at various taxonomic levels (S3 Fig). Interaction network analysis further substantiated that the interaction of fungal genera in the mycobiomes of healthy controls, T2DM and DR are distinct and different (S4 Fig).

Alpha diversity changes in gut mycobiomes in individuals in the diseased state compared to the healthy individuals indicated mixed trends in the Alpha diversity indices. The observed number of OTUs and Chao1 index were significantly reduced only in DR mycobiomes compared to HC mycobiomes and this agrees with earlier observations that mycobiomes could exhibit reduced diversity as in paediatric Inflammatory Bowel Disease (IBD) [43], in anorexia nervosa [42], in obese subjects [41] and Ulcerative Colitis Patients [29]. Additionally, the diversity indices in DR were significantly reduced when compared to T2DM microbiomes. In partial agreement with earlier studies that indicated increased richness in patients with hepatitis B [37] and Crohn’s disease (CD) in adults [26, 31], we report increased richness and evenness in T2DM compared to HC mycobiomes. Further in a few cases α-diversity indices did not differ significantly as in children with CD [23] and in T1DM patients [40], but we consistently observed that more than one parameter differed significantly in T2DM and DR mycobiomes. Further, it is not easy to compare the results across the studies since not all the mycobiomes studies have provided data on all the indices. Our studies on fungal gut microbiomes related to ocular diseases like bacterial keratitis (BK), fungal keratitis (FK) and Uveitis (UVT) indicated mixed trends in the Alpha diversity indices such as: (i) HC and FK mycobiomes exhibited equivalent number of observed OTUs and had similar Shannon (diversity) and Simpson indices (evenness) [19], (ii) in BK individuals the mycobiomes showed increase in Shannon index and Simpson index but a decrease in number of OTUs and Chao1 index (richness) [18] and (iii) in UVT individuals the mycobiomes showed similar Shannon and Simpson indices whereas increase was observed in number of OTUs and Chao1 index compared to HC [59]. Thus drawing a generalized conclusion about the correlation between diseases and fungal diversity is difficult. At the moment we do not have a plausible explanation.

### Factors that could influence the gut mycobiomes

Diabetes, different blood sugar levels or anti-hypertension drugs could have direct modulatory effects on the gut microbiome. Thaiss et al. [87] demonstrated using a mouse model of type 1 diabetes mellitus that high blood sugar (hyperglycemia) causes a leaky gut barrier and changes the gut microbiota. This observation contradicts the work by Cani and Delzenne [88] who proposed that the diet caused dysbiosis and barrier dysfunction and as a consequence bacterial endotoxins pass through a leaky gut barrier and drive low grade inflammation. Such an inflammation could be the cause of glucose intolerance and elevated blood sugar in patients. Recent studies have also indicated that proton pump inhibitors, metformin, selective serotonin reuptake inhibitors and laxatives influence gut microbiome composition and function [89]. For instance changes in the gut microbiome following proton pump inhibitor has been associated with enteric infections, including *Clostridium difficile* infections, with anti-tumour response and alterations in drug bioavailability, bioactivity or toxicity. Though data is available on bacterial microbiomes their influence on the mycobiome are lacking. In the present study individuals with the above factors and other co-morbidities like Inflammatory bowel disease (IBD), paediatric IBD, Crohn’s disease (CD), Irritable Bowel Syndrome (IBS), Pancreatic Ductal Carcinoma (PDC) and Colorectal Cancer (CC) which may have modulatory effects on the mycobiome were also excluded.

This study does not provide insight into the mechanism of how changes in the gut mycobiome influences DR. But it could be similar to gut microbiome activating UVT wherein Uveitis-relevant cells, the TH17 cells in the intestine reach the eye to cause UVT [8, 90]. Another possibility is that dysbiosis may be modulating growth factors like VEGF (vascular endothelial growth factor) implicated in retinopathy [91]. It is known that gut microbiota regulates VEGF in intestinal macrophages [92].

## Conclusions

- This is the first study demonstrating that the gut mycobiomes of HC, T2DM and DR could be discriminated at the phyla and genera level by heat map, Beta diversity analysis using NMDS plots and LEfSE analysis.
- The data could help in developing novel therapies for treatment of DM and DR based on the functional attributes of the discriminating fungi.
- Research unravelling the functions of the discriminating fungal genera in longitudinal studies and by sampling across ethnicities would strengthen attempts at using fungi as therapeutic agents.

## Limitations

1. Longitudinal studies involving individuals when first diagnosed with DR would help to identify microbial dynamics with progression of the disease.
2. Involving more individuals across geographical regions may unravel ethnic differences in mycobiome in the DR diseased state.

## Supporting information

S1 Fig Rarefaction analysis of fungal diversity in the gut mycobiomes of healthy controls (HC, n = 30), Type 2 Diabetes Mellitus (T2DM, n = 21) and Diabetic Retinopathy (DR, n = 24) individuals. (TIF)

S2 Fig Alpha diversity indices after rarefying the library size (Shannon diversity index, Simpson index, number of observed OTUs, and Chao1 index). Shannon and Simpson indices were statistically significant between HC and T2DM (indicated by ★) and observed number of OTUs and Chao1 index were statistically significant between HC and DR (indicated by ★★) and T2DM and DR (indicated by **#**) (*P* = < 0.05).
(TIF)

S3 Fig Cladoplots depicting differential microbial features of HC, T2DM and DR selected by linear discriminant analysis effect size analysis. Differential taxa between HC, T2DM and DR are depicted in different color for the most abundant class: green indicating increase in HC, blue indicating increase in T2DM and red indicating increase in DR patients.
(TIF)

S4 Fig Significant co-occurrence and co-exclusion relationships at genus level in the gut mycobiomes of healthy controls (HC, n = 30), Type 2 Diabetes Mellitus (T2DM, n = 21) and Diabetic Retinopathy (DR, n = 24) patients. **(A)** Interaction of fungal genera in the gut mycobiomes of healthy controls (HC), **(B)** Type 2 Diabetes Mellitus (T2DM) and **(C)** Diabetic Retinopathy (DR) patients. The size of the nodes in the network corresponds to their degree of interaction. The positive and negative correlations/interactions are indicated with green and red edges respectively.
(TIF)

S1 Table Diet and clinical characteristics of HC, T2DM and DR patients. (XLSX)

S2 Table Relative abundance of fungal OTUs for 75 fungal mycobiomes. Sparse OTUs (with < 0.001% of total number of reads assigned to OTUs) were not included.
(XLSX)

S3 Table Number of OTUs assigned at phylum level. (XLSX)

S4 Table Relative abundance of fungal phyla in the studied cohort. (XLSX)

S5 Table Number of OTUs assigned at genus level. (XLSX)

S6 Table Relative abundance of fungal genera in the studied cohort. (XLSX)

S7 Table Median abundance (%) of fungal genera for 75 fungal mycobiomes. Sparse OTUs (with < 0.001% of total reads assigned to OTUs) were not included.
(XLSX)
