# Supplementary figures and images for: Gut mycobiomes are altered in people with type 2 Diabetes Mellitus and Diabetic Retinopathy
Source: PLoS One. 2020 Dec 1;15(12):e0243077. doi: 10.1371/journal.pone.0243077 (PMC7707496; doi:10.1371/journal.pone.0243077)

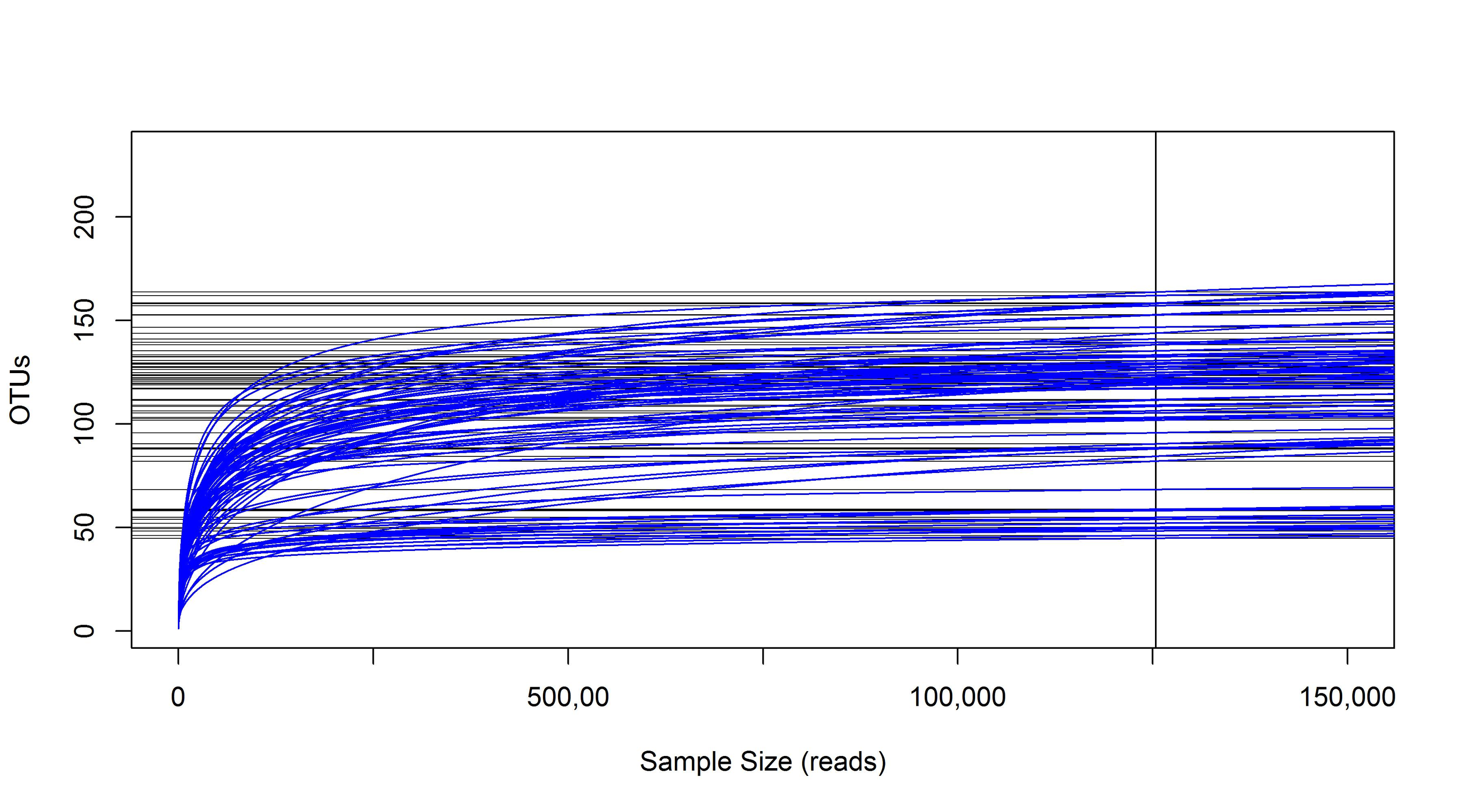

Supplement: S1 Fig — (TIF) [file pone.0243077.s001.tif]

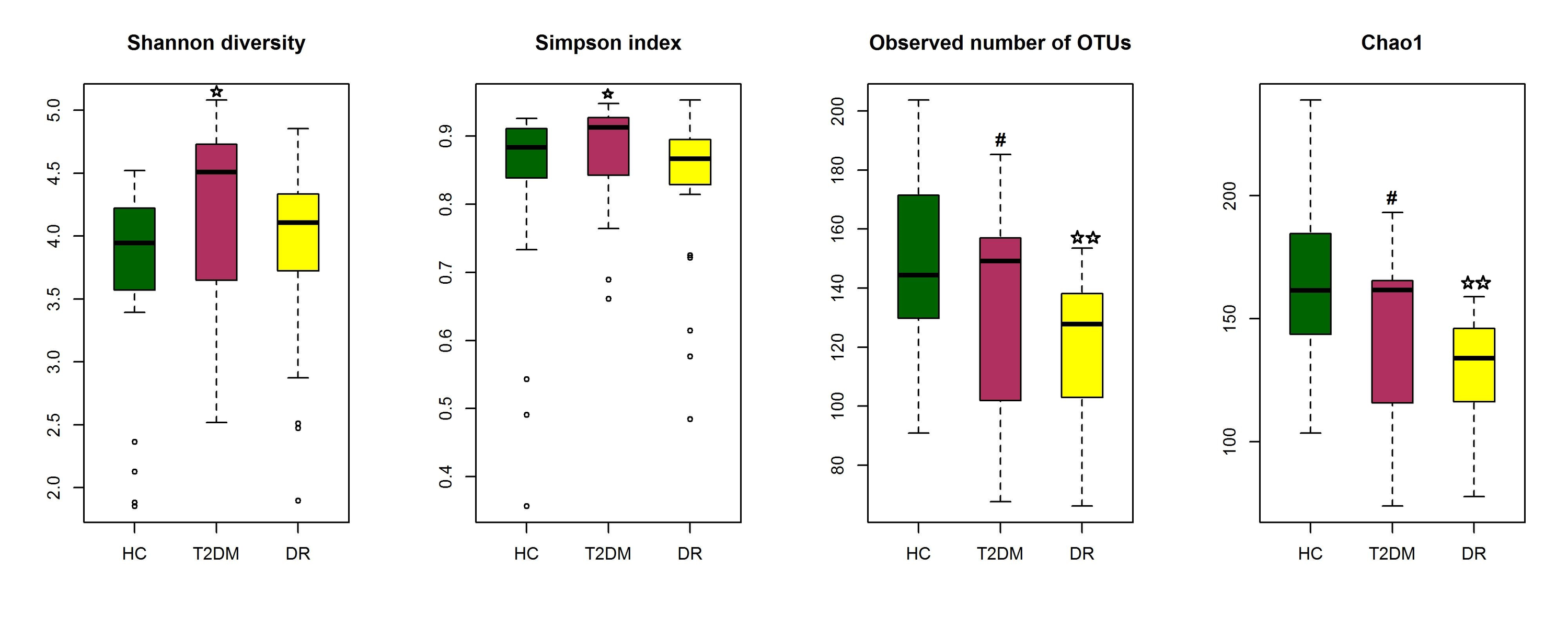

Supplement: S2 Fig — Shannon and Simpson indices were statistically significant between HC and T2DM (indicated by ★) and observed number of OTUs and Chao1 index were statistically significant between HC and DR (indicated by ★★) and T2DM and DR (indicated by #) (P = < 0.05). (TIF) [file pone.0243077.s002.tif]

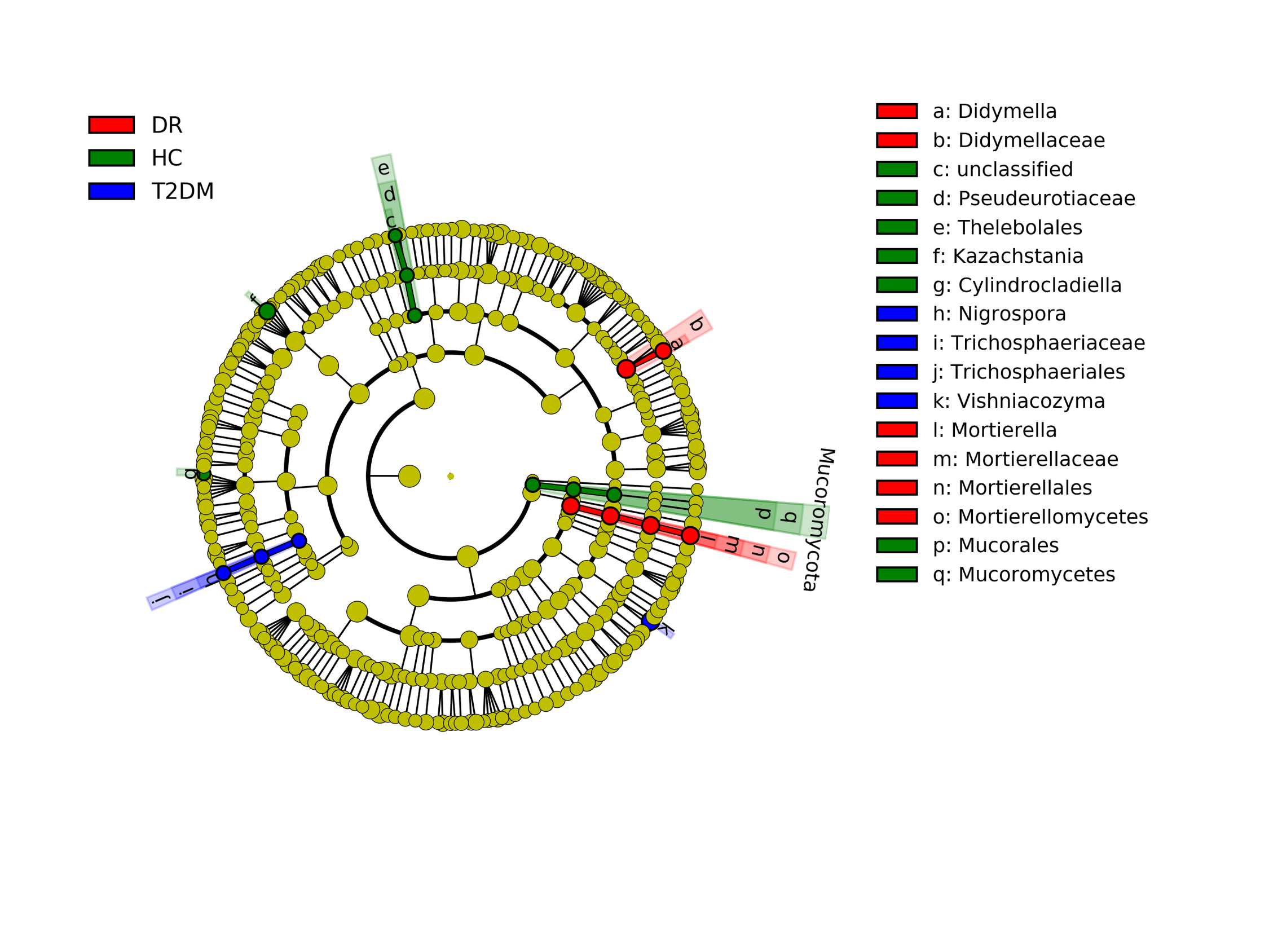

Supplement: S3 Fig — Differential taxa between HC, T2DM and DR are depicted in different color for the most abundant class: green indicating increase in HC, blue indicating increase in T2DM and red indicating increase in DR patients. (TIF) [file pone.0243077.s003.tif]

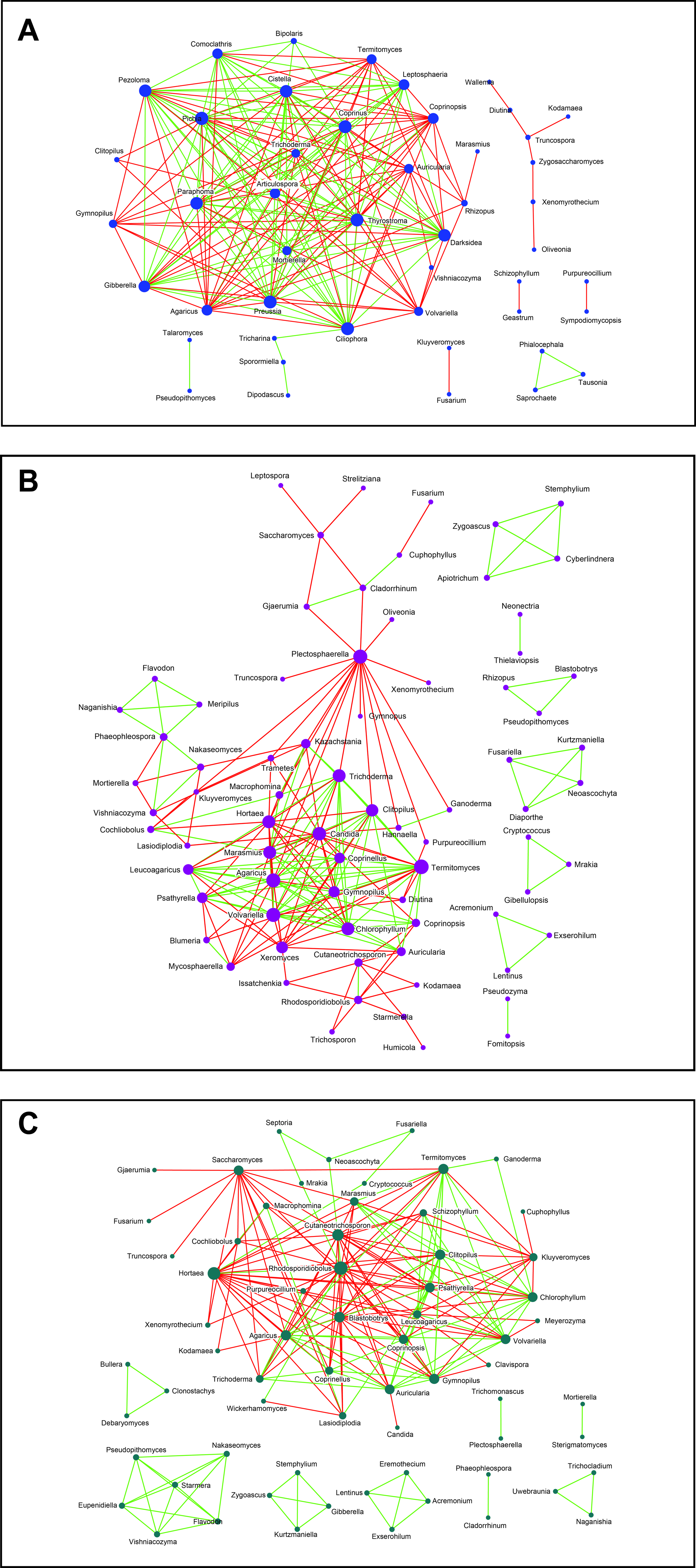

Supplement: S4 Fig — (A) Interaction of fungal genera in the gut mycobiomes of healthy controls (HC), (B) Type 2 Diabetes Mellitus (T2DM) and (C) Diabetic Retinopathy (DR) patients. The size of the nodes in the network corresponds to their degree of interaction. The positive and negative correlations/interactions are indicated with green and red edges respectively. (TIF) [file pone.0243077.s004.tif]
